# The Histaminergic Tuberomamillary Nucleus Is Involved in Appetite for Sex, Water and Amphetamine

**DOI:** 10.1371/journal.pone.0148484

**Published:** 2016-02-04

**Authors:** Marco Contreras, María E. Riveros, Maricel Quispe, Cristián Sánchez, Guayec Perdomo, Fernando Torrealba, José L. Valdés

**Affiliations:** 1 Departamento de Ciencias Fisiológicas, Facultad de Ciencias Biológicas, Pontificia Universidad Católica de Chile, Alameda 340, Santiago, Chile; 2 Programa disciplinario de Fisiología y Biofísica, Biomedical Neuroscience Institute, BNI, I.C.B.M., Facultad de Medicina, Universidad de Chile, Independencia 1027, Santiago, Chile; 3 Centro de Fisiología Celular Integrativa, Facultad de Medicina, Clínica Alemana Universidad del Desarrollo. Santiago, Chile; Medical University of South Carolina, UNITED STATES

## Abstract

The histaminergic system is one component of the ascending arousal system which is involved in wakefulness, neuroendocrine control, cognition, psychiatric disorders and motivation. During the appetitive phase of motivated behaviors the arousal state rises to an optimal level, thus giving proper intensity to the behavior. Previous studies have demonstrated that the histaminergic neurons show an earlier activation during the appetitive phase of feeding, compared to other ascending arousal system nuclei, paralleled with a high increase in arousal state. Lesions restricted to the histaminergic neurons in rats reduced their motivation to get food even after 24h of food deprivation, compared with intact or sham lesioned rats. Taken together, these findings indicate that the histaminergic system is important for appetitive behavior related to feeding. However, its role in other goal-directed behaviors remains unexplored. In the present work, male rats rendered motivated to obtain water, sex, or amphetamine showed an increase in Fos-ir of histaminergic neurons in appetitive behaviors directed to get those reinforcers. However, during appetitive tests to obtain sex, or drug in amphetamine-conditioned rats, Fos expression increased in most other ascending arousal system nuclei, including the orexin neurons in the lateral hypothalamus, dorsal raphe, locus coeruleus and laterodorsal tegmental neurons, but not in the ventral tegmental area, which showed no Fos-ir increase in any of the 3 conditions. Importantly, all these appetitive behaviors were drastically reduced after histaminergic cell-specific lesion, suggesting a critical contribution of histamine on the intensity component of several appetitive behaviors.

## Introduction

The increase in arousal state during the appetitive phase of motivated behaviors such as feeding, exploration, drinking or mating is a necessary brain state for the adequate execution of these behaviors. The increase in arousal is recognizable by a rise in sensory responsiveness, locomotion, and catabolic vegetative activity [1–3]. Previous works have shown early activation of histaminergic neurons during the appetitive phase of feeding, even before the involvement of other ascending activating system (AAS) nuclei [4]. Lesion of histaminergic neurons abolished the behavioral and vegetative arousal, the general cortical activity and the activity of other components of the AAS induced by food enticing [5]. Exploratory activity in new environments (another behavior that could be considered a motivated behavior) is also disrupted in mice lacking the histamine-synthesizing enzyme or the histamine H1 receptor [6,7]. However, it is still unknown if the histaminergic system is also necessary for motivated behaviors different from feeding or spontaneous exploratory behavior, which would indicate that the histaminergic system plays a more widespread role in the regulation of the arousal state during goal-directed behaviors.

To answer this question we studied the pattern of AAS nuclei activation during the appetitive phase of sexual, water, or drug-related behaviors using c-Fos immunoreactivity [8] and the change in these appetitive behaviors in rats with cell-specific lesion of the histaminergic tuberomamillary nucleus (TMN) [9]. We used male rats in three experimental paradigms: (1) exposure to receptive females, allowing sensory contact (olfactory, visual) while preventing mating; (2) enticing thirsty rats (48 h water deprivation) with an empty drinking bottle and (3) the change in place preference of rats conditioned during 10 days to receive daily amphetamine injections, after 10 additional days of amphetamine withdrawal [10]. In all cases, the consummatory phase of the behavior was prevented in order to prolong and isolate the appetitive component of the behavior. This separation between appetitive and consummatory phases has been proposed by Sherrington 1906 [11] and Craig 1917 [12], and recently revisited by Ball and Balthazart [13], and it is critical to understand the different neural substrates of these behavioural stages.

## Materials and Methods

### Subjects

A total of 127 male and 10 female Sprague-Dawley rats were used. Animals weighing 270 to 350 grams were kept in separate cages under controlled temperature (21–24°C) and 12/12 hours, light/dark schedule. Water and food were supplied *ad libitum*, unless other condition was indicated. Males and females were individually housed in separate rooms to prevent male perception of the female incentives prior to testing. All experiments were carried out in accordance to the NIH Guide for the Care and Use of Laboratory Animals (NIH Publication No. 80–23, revised 1996), minimizing the number of animals used and their suffering. All the experimental protocols were approved by our local institutional Bio Safety and Bio-Ethical Committee of the Faculty of Biological Sciences, P. Universidad Católica de Chile.

### Sexual appetitive behavior

Female sexual receptivity was determined according to the phase of the estrus cycle, assessed by daily vaginal smears [14]. Only females that showed at least two regular 4-days cycles were used. Each participating female was either in proestrus (n = 5) (sexually receptive) or in diestrus (n = 5) (non-receptive). Male rats without previous sexual experience (n = 10) were exposed during 30 minutes to one sexually receptive female or to one non-receptive female by using an acrylic box (47cm. x 28cm. x17cm.) divided into two compartments by a transparent acrylic panel with multiple 1 cm in diameter holes (separated equidistantly by 1 cm each one) that prevented the copula, but allowed visual, auditory, and olfactory contact. Naïve males displayed intense sniffing oriented to receptive females but not to non-receptive females, in agreement with previous studies where appetites were induced by odor from feces of female rats in oestrus. Naïve or experienced males showed penile erections and an increase in exploration directed to the odor container.[15]. Nineteen TMN lesion rats were exposed to receptive female in the same way. Before exposure to the females, the males were habituated to the box for 2 hours during 4 consecutive days. After each experiment, the boxes were thoroughly rinsed with distilled water. To determine the magnitude of male sexual motivation we measured the time that male rats spent sniffing through the holes in the panel during the first 30 minutes of exposure to the female.

### Appetitive behavior directed to obtain water

Male rats were deprived (“deprived” group) of water for 48h and then anesthetized with chloral hydrate (350 mg/Kg, i.p.) and transcardially perfused with fixative after the deprivation period (n = 6).We noted, in agreement with Dufort & Abrahamson [16], that a water deprivation of 24h was not enough to elicit consistent or maximal appetitive behavior. Although it is documented that 24 h of deprivation induce a 2.2% of increase in the plasma osmolality, which is just over the threshold for thirst (1.6%) in rats [17], a 48 h deprivation period was required to elicit the maximum response. A second group (n = 14) was also water deprived for 48 h, and euthanized by the same procedure, but after 30 min of enticing them (“deprived enticed” group) with an empty water bottle placed on their home cages. The same 19 TMN lesion rats were tested for water appetitive behavior. The motivation for water was evaluated by measuring the time the rat spent licking the spout of the empty water bottle.

### Amphetamine appetitive behavior: place preference conditioning

We used a two-compartment biased apparatus to assess place preference [10]. The apparatus consisted of a smaller central alley (40cm. x 30cm. x 25cm.) with dark brown walls, connecting two square compartments of similar size (45 cm x 45 cm x 45 cm); one had white walls and the other had black walls. The rats were placed at the center of the connecting alley and video recorded with a zenithal digital camera for 5 minutes, to assess spontaneous place preference. As shown previously [10], the rats tend to avoid the bright, white-walled compartment, and stay in the dark, black-walled room, following their natural avoidance of illuminated spaces. A group of rats with a TMN lesion (n = 12, different from those used for water and sex) received a dose of D-amphetamine (1.5 mg/Kg, a kind donation of Laboratorios Chile S.A., Santiago, Chile dissolved in 1 ml of sterile saline) while in the non-preferred (white) compartment. They were left there for 30 minutes and then returned to their home cages. On alternate days, the rats received an injection of 1 ml of saline and were left in the black compartment for 30 minutes; this treatment lasted for 10 days. A second group of intact rats (n = 6) received the same treatment. The third group consisted of intact rats that received a daily injection of saline (n = 6), one day in the white and the next day while they stayed in the dark compartment, also for 10 days.

After the 10 days of conditioning, the 3 groups had 10 additional days of rest, and were then tested for place preference for 30 minutes. During the first 10 minutes, the time spent on each compartment was recorded, and changes with respect to the initial preference were computed. Rats were considered conditioned when the time spent in the amphetamine-paired compartment during the test was 2SD over the mean time spent in the same compartment by control animals injected with saline.

### Evaluation of neural activity in the AAS neurons

Immediately after the 30 minutes of sex, water, or amphetamine enticing the rats were anesthetized and processed for histology, as detailed below, to evaluate Fos-ir changes in the AAS. We chose 30 minutes after the onset of the appetitive behavior because that was the earliest time we found significant changes in Fos-ir among AAS nuclei [4] during enticement with food. If instead of 30 minutes we had waited for 60 or 90 min before anesthesia and perfusion, we would not be able to discriminate the earliest activation in the AAS nuclei, as shown before [4]. To control for baseline Fos-ir, we used the brains of 15 naïve male rats from our collection, which were euthanized at the same clock times of the experimental groups, but were not under any experimental manipulation; this control group is intended as a circadian control of Fos-ir of selected structures.

### TMN lesions

We used orexin B coupled to the ribosomal toxin saporin (OrxB-SAP) (Advanced Targeting System, San Diego, CA, USA) [5,9], to make a cell specific lesion of the TMN. TMN and some neighboring neurons that express type 2 orexin receptor are readily damaged by this toxin. Thirty nine rats received bilateral injections of 50 ng/0.5 μl of OrxB-SAP, using glass micropipettes connected to a pneumatic picopump and stereotaxic guidance, aiming at the TMN, at the following coordinates: -4.3 mm from bregma, 1.2 mm laterality and 9 mm in depth from the cortical surface [18]. Recovery time before experimental procedures was 1 week. We counted ADA-ir (Adenosine Deaminase, a marker of histaminergic neurons [19]) neurons in three sections of the dorsal and ventral part of TMN. Rats were considered as having a lesion (31 out of 39) when the number of ADA-ir neurons in the dorsal and/or the ventral part of TMN was lower than the mean minus 2SD of the number of neurons computed in the intact control group (191.6 ADA-ir neurons/mm^2^ in the TMN ventral part and 196.5 ADA-ir neurons/mm^2^ in the TMN dorsal part). The average of ADA-ir neurons in the intact control group which was 284.8 neurons/mm^2^ for ventral TMN and 217.3 neurons/mm^2^ in the dorsal part will be used as 100% of ADA-ir neurons to compute the percentage of lesion. Additionally, two adjacent neural populations (Orexin neurons in the lateral hypothalamic/perifornical area (LHA) at -3.6mm from bregma and ventral tegmental area (VTA)/TH-ir neurons at -5.0mm from bregma) were counted to control for the possible spread of the toxin [5]. An intact non-lesioned animal group (n = 20) was used as control. No additional sham lesioned group was included in the present work, since we previously demonstrated that sham lesioned animals exhibit no difference in locomotor activity, body core temperature, circadian rhythms, motivation to get food, or c-fos patterns of activity [5]. Animals that did not reach the criteria for lesion were not included in the present work because of the high degree of variability in their behavior.

### Histology

At the end of the experiments, all rats were deeply anesthetized with chloral hydrate (350 mg/Kg; i.p.) and transcardially perfused with a saline flush followed by 500 ml of 4% paraformaldehyde in phosphate buffered saline (pH 7.4). The brains were post-fixed in the same fixative for 2 h and transferred to 30% sucrose with 0.02% sodium azide in PBS until they sank. Brains were cut in the coronal plane, at 50 μm thickness, using a sliding frozen microtome. The sections were processed for Nissl staining and immunohistochemistry.

### Immunohistochemistry

Free-floating sections were incubated overnight at room temperature in the primary antibody (Fos polyclonal antibody, Ab-5 rabbit polyclonal, from Oncogene, San Diego, CA, diluted 1:20,000), incubated for 1 hour in the secondary antibody (Biotin-SP- conjugated AffiniPure goat anti-rabbit IgG H+L; from Jackson ImmunoResearch, PA, diluted 1:1,000), rinsed, and incubated for 1 hour in Vectastain ABC Elite Kit (Vector Laboratories, CA, diluted 1:500). Finally, the sections were revealed with 0.05% 3–3’diaminobenzidine hydrochloride (DAB) and nickel chloride, producing an enhanced dark blue reaction product. Selected sections already immunostained for nickel-enhanced Fos-ir were subjected to a second immunostaining to identify ADA-ir neurons in the TMN, or tyrosine hydroxylase (TH)-ir neurons in the VTA and locus coeruleus, or orexin-ir neurons in the LHA. The second immunostaining was revealed with DAB without nickel intensification, yielding a brown cytoplasmic precipitate that contrasted with the dark blue nuclear DAB-nickel labeling of the Fos-ir. The antisera used were anti ADA (polyclonal, raised in rabbit, diluted 1:5,000 from Chemicon, CA); anti-orexin A (rabbit polyclonal, 1:20,000; from Phoenix Pharmaceutical Inc., CA) and anti-TH (rabbit polyclonal, 1:10,000; from Chemicon, CA). Specificity of these antibodies was tested before [4].

### Quantification of Fos-ir

The Fos immunoreactivity of the different nuclei of the AAS was assessed by counting Fos-ir neurons bilaterally when correspond, in 3 evenly spaced coronal sections from each nucleus, per rat as described previously [4]. In brief, in the TMN and the lateral hypothalamic area we counted the neurons doubly labeled for Fos-ir and either ADA-ir or orexin-ir, respectively. For the locus coeruleus (LC) and VTA area we used TH-ir sections to identify this structure but only counted Fos-ir neurons by using grids of appropriate size, due to the difficulty in delimiting single TH-ir neurons. Dorsal raphe (DR) and laterodorsal tegmental nuclei were identified by Nissl staining, and Fos-ir neurons were counted, no additional markers where used for this magnocellular nuclei, however they are easily identifiable in the co-staining of Nissl and Fos-ir, as it was described in detail before [4].

### Statistic

The number of Fos-ir neurons on each nuclei, for each conditions was compared by using a one way ANOVA of Ranks (Kruskall Wallis) followed by a post-hoc of multiple comparison, Dunn's method. The comparison of neurons per section in the LHA and VTA region as control of the spare of the ribosomal toxin was conducted by using a Mann Whitney test and the behavioral responses were compared by using a one way ANOVA of Ranks (Kruskall Wallis) followed by a post-hoc of multiple comparison, Dunn's methods with a significance level of p<0.05 in all test used. All the statistical analysis was conducted by using the SigmaStat 3.0 sofware (SPSS, Chicago, IL, USA).

## Results

### AAS activation during the appetitive phases of sex, water drinking and drug related behaviors

**Fig 1:**
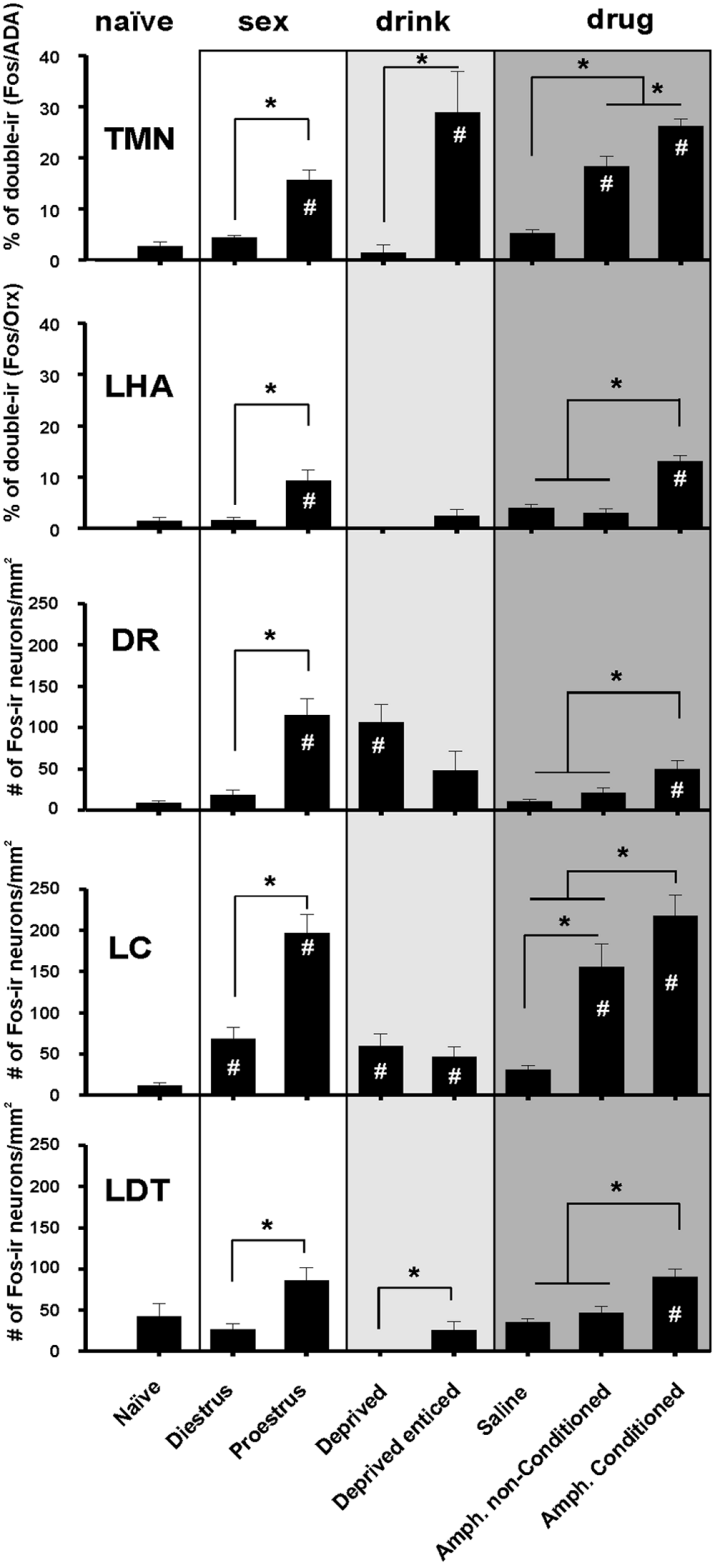
Fos-ir pattern of AAS nuclei elicited by appetitive behavior. Left column in white (sex): Fos-ir after 30 min of exposure to receptive (proestrus n = 5) or to non-receptive (diestrus n = 5) females. Middle panel in light gray (drink): 48 hours of water deprivation followed by 30 minutes of enticing (Deprived enticed n = 14) or not (Deprived n = 6). Right column in dark gray (drug): 30 minutes of exposure to the place preference apparatus where the animals were conditioned to amphetamine (Amph. Conditioned), versus non-conditioned animals (Amph. non-Conditioned) and rats non-conditioned injected with saline, (saline). # p< 0.05 respect to the naïve circadian controls; * p< 0.05 between conditions. Kruskall Wallis one way ANOVA followed by all pairwise multiple comparisons (Dunn's Method).

The exposure of male rats to a receptive female (proestrus) significantly increased the number of Fos-ir neurons in all nuclei of the AAS respect to the naïve circadian control group (Fig 1, left white column under sex). In contrast, the exposure to non-receptive (diestrus) females did not increase the number of Fos-ir neurons of the AAS, except for the LC.

Water deprivation *per se* increased Fos-ir in the DR and LC (Fig 1, middle column in light gray, "drink") relative to naïve rats. Thirsty rats enticed with an empty drinking bottle had a large and significant increase in the number of Fos-ir neurons in the TMN. No further increase was observed in the LC or DR. It was revealing that the enticing with the empty water bottle, but not thirst by itself, induced Fos-ir only in the TMN.

**Fig 2:**
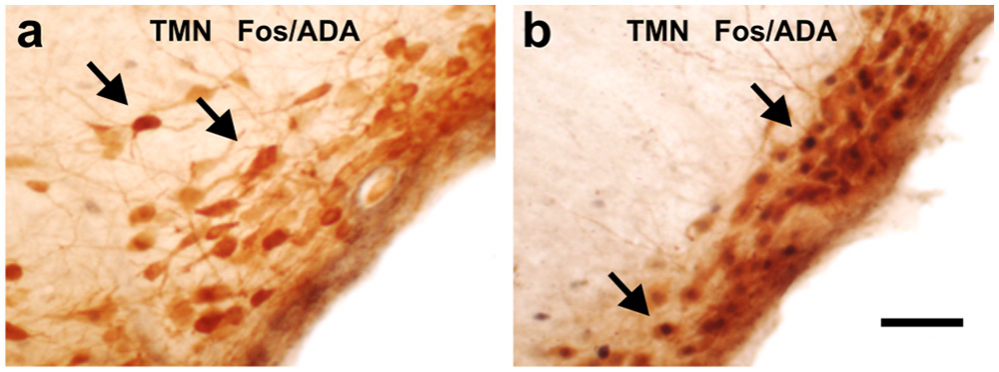
Photomicrograph of TMN neurons after re-exposure to the amphetamine place preferences apparatus. **(a)** Double immunohistochemistry for Fos/ADA in the tuberomamillary nucleus of a naïve (circadian control) rat. **(b)** Double immunohistochemistry for Fos/ADA in the TMN of an amphetamine conditioned rat, after 30 minutes of re-exposure to the amphetamine place preference apparatus. Arrows in **(a)** depict ADA-ir neurons; arrows in **(b)** depict double-ir neurons Fos/ADA. Scale bar, 200 μm.

During the place preference test and after re-exposure to the conditioning apparatus, we compared the activity of the AAS nuclei in animals conditioned to amphetamine with those which were not. Animals injected with saline rather than amphetamine showed no increase in Fos-ir in the AAS nuclei with respect to the naïve control group (Fig 1, right column in dark gray, "drug"). Rats treated with daily amphetamine, but that did not reach the conditioning criteria (see methods), showed activation of the TMN and LC. However those rats that were conditioned to amphetamine showed a significantly higher activation of the TMN (Fig 2a and 2b), LC, and of orexin-ir neurons from the lateral hypothalamic area (Orx/LHA); DR and lateral dorsal tegmental (LDT) nuclei were also engaged.

In summary, the TMN was active during the appetitive phase of the three motivated conditions, whereas the LHA/Orx and LDT neurons were engaged in sexual appetite and drug enticing, but not in the drinking water paradigm. DR and LC were engaged during sex or drug appetitive behaviors. The VTA showed no Fos-ir increase in any of the 3 conditions, S1 Fig. It is worth noting that only the TMN was active in drinking or feeding [4] appetitive behaviors, probably because they are more crucial to survival.

### TMN lesion histology

**Fig 3:**
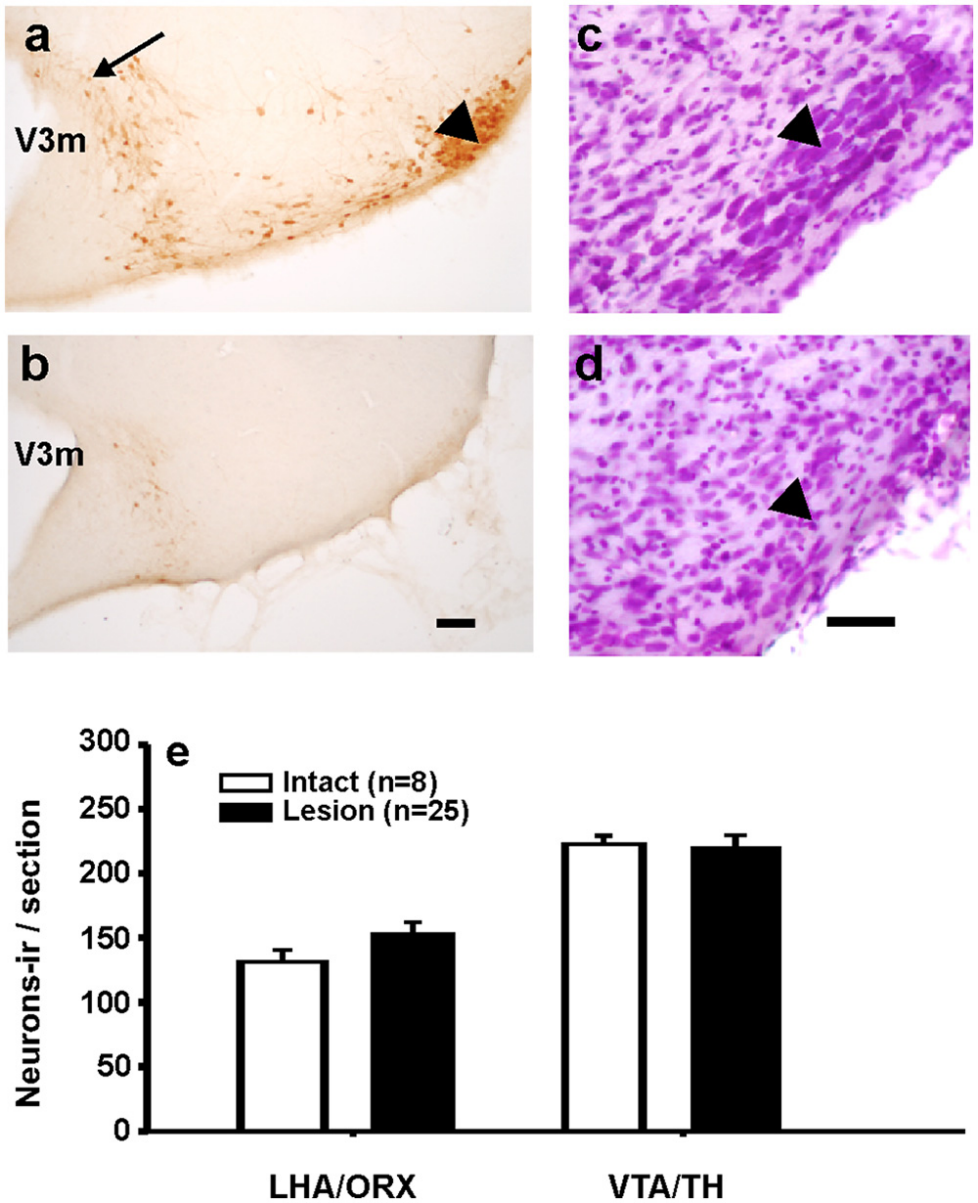
Quantification of the OrxB-SAP ribotoxin lesion of the TMN and adjacent structures. Photomicrographs of sections stained for ADA-ir (**a, b**) and Nissl substance (**c, d**). **(a)** and **(c)** show an intact TMN, (**b**) and (**d**) represent a large TMN lesion (77%) that involved the ventral (arrowhead) and the dorsal TMN (arrow). **(e)**, the quantification of Orx-ir neurons in the LHA or TH-ir neurons in the ventral tegmental area (VTA), demonstrated that these adjacent regions that express Orx receptors were spared by the Orx-SAP injections. No statistical differences were detected (Mann Whitney test, p = 0.216 and 0.811 respectively). Scale bars = 200 μm. **V3m** mammillary recess of the third ventricule.

Lesion of histaminergic neurons was successful in 31 of 39 rats, according to the criterion described above. The percentage of lesion ranged from 4% to 77% of the total, considering 100% as the average number of neurons observed in intact rats. Microphotographs of the TMN region stained for ADA immunohistochemistry and Nissl substance are depicted in Fig 3 of intact (Fig 3a and 3c) or lesioned (Fig 3b and 3d) rats. The lesioned rats clearly showed an absence of ADA-ir neurons (Fig 3b) and, in the Nissl staining (Fig 3d), a complete loss of magnocellular neurons (black arrowhead), without the presence of additional tissue damage or gliosis. Regions adjacent to the TMN nucleus, such as TH-ir neurons in the VTA or Orx-ir neurons in the lateral hypothalamic area (Fig 3e), were not affected by the ribotoxin, as it was also demonstrated before [5].

### Behavioral effects of the TMN lesion

**Fig 4:**
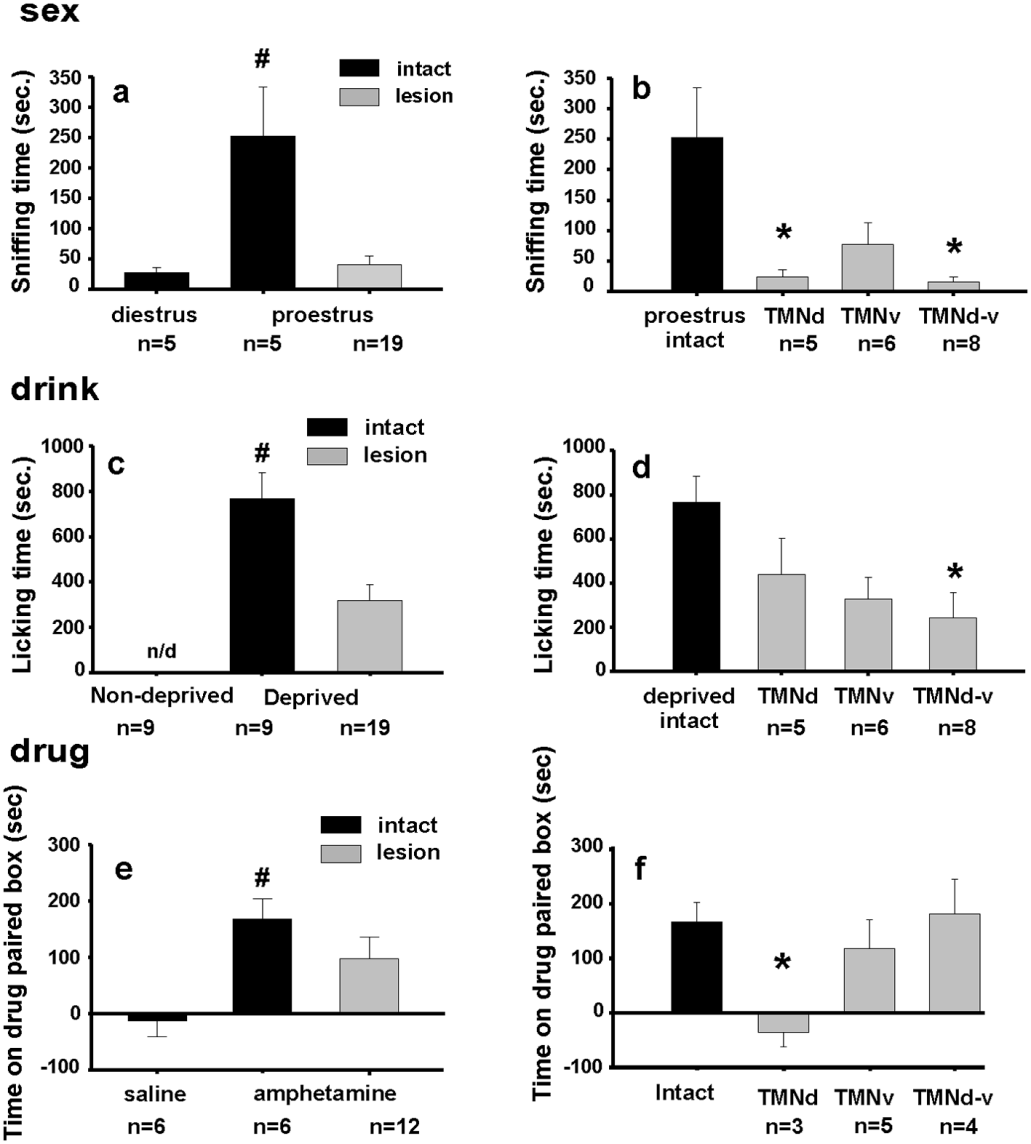
Effect of TMN lesion on appetitive behavior. (**a)** TMN lesion decreased the sniffing time of male rats challenged by a receptive female (proestrus) to a level similar to that elicited by non-receptive females (diestrus).(**b)** dorsal TMN and dorsal plus ventral TMN lesions were sufficient to reduce sexual appetitive behavior respect to the intact group. (**c)** TMN lesion decreased licking time of water deprived animals enticed by an empty drinking bottle. (**d)** The lesion had to involve both TMN divisions to be effective on drinking behavior. (**e)** TMN lesion decreased the time spent in the amphetamine-paired box in conditioned rats. (**f),** rats with a dorsal TMN lesion showed the largest drop in appetitive behavior directed to obtain amphetamine. ANOVA of Ranks (Kruskall Wallis) followed by a post-hoc of multiple comparison, Dunn's method. * indicates p<0.05 respect to intact rats, # p<0.05 respect to the two other conditions. n/d, not detected.

The appetitive behaviors induced by sexual drive, thirst or amphetamine conditioning were decreased after the TMN lesion (Fig 4).

The sniffing sexual behavior elicited by the receptive females (Fig 4a) was observed in the intact animal group, but totally abolished in TMN lesion animals. Splitting the lesion group according to which TMN region was significantly compromised by the ribotoxin, we observed that sexual appetitive behavior was more influenced by the dorsal TMN lesion (Fig 4b).

Drinking behavior was more compromised after extensive TMN lesion (dorsal and ventral, (Fig 4c and 4d); note that the same rats were used to test sex and drinking behaviors.

In contrast, drug-seeking behavior was more affected by a dorsal TMN lesion (Fig 4e and 4f). In this case the percentage of lesion in the pure dorsal lesion group was 44.15% and in the pure ventral group was 42.82%. Then in the dorsal-ventral lesion group, even though the degree of lesion in the ventral part was equivalent to the pure ventral (42.02%), the lesion of the dorsal component was only 23.59%, indicating that the behavioral effect is expressed after a high compromise of the dorsal component of TMN exclusively. Taken together, the results of these lesions suggest a differential functional topography inside the TMN nucleus in relation to different appetitive behaviors.

Linear regression was significant and positive between lesion size of the TMN as a whole and drinking, or between dorsal TMN and drug-related behavioral responses, see Table 1.

**Table 1:** Linear regression between TMN lesion extension and behavior during the appetitive phase of sex, drink and drug motivated behaviors.

TMN Lesion			
	Total	Dorsal	Ventral
**Sex**			
r	0.106	0.221	0.047
p	0.667	0.489	0.847
n	19	13	14
%	27.36 +/- 4.97	25.13 +/- 5.22	43.22 +/- 6.16
**Drink**			
r	*0*.*502*	0.383	0.380
**p**	***0*.*042***	0.129	0.223
n	*17*	13	14
%	*27*.*49 +/- 5*.*55*	43.58 +/- 6.85	26.05 +/- 5.41
**Drug conditioned**			
r	0.076	*0*.*791*	0.388
p	0.815	***0*.*034***	0.242
n	12	7	9
%	19.06 +/- 3.86	32.40 +/- 4.98	36.07 +/- 5.24

The r and p values of the regression, the number of animals (n) and the % of the lesion, are indicated. Significant regressions are in bold.

The lesion experiments strongly suggest that the TMN is necessary for a variety of appetitive behaviors, either alone (drinking or feeding) or together with other AAS nuclei (sex and drug).

## Discussion

The present findings support in two ways the hypothesis that the histaminergic system plays a necessary role in the regulation of the appetite phase of different goal directed behaviors. First, we show here that an early increase in the neural activity of histaminergic neurons characterizes different motivated behaviors. While each of these behaviors has a specific pattern of AAS nuclei activation, it always includes the activation of the TMN. Secondly, TMN lesion prevented the correct unfolding of appetitive behaviors related to feeding [4], thirst, sex, or amphetamine conditioning.

While all AAS nuclei other than the VTA were active during sexual or amphetamine-related appetitive behaviors, during drinking appetitive behavior only the TMN was active at earlier times, similar to the case of feeding, as shown before [4]. It is important to recall here that, when Fos-ir is assessed 30 minutes after the initiation of an appetitive behavior such as feeding [4], hungry rats in a state of a high arousal induced by presentation with inaccessible food show a significant initial activation of the histaminergic neurons. Additional activation of other AAS nuclei was only observed when the enticing was prolonged to 60 minutes.

The validity of arousal as a single construct has been strongly questioned [3,20] on several grounds. In addition to the existence of several components of the arousal system (aminergic nuclei and orexin/hypocretin neurons), experimental interference with individual aminergic nuclei from the AAS causes specific cognitive or behavioral deficits. Systematic studies [3] have shown that the outputs of these nuclei are not functionally interchangeable. Our claim here, also made by Robbins and Everitt [3], is that the inputs that activate each of the arousal nuclei are also specific, and so these nuclei are not engaged as a whole to increase arousal in every behavioral situation. Rather, each of them contributes to particular behaviors in response to specific inputs, producing unique (also because their outputs are distinctive) arousal responses. For instance, it has been proposed that the activation of serotoninergic neurons is related to arousal and anxiety [21] while LC activity modulates sensory responses and attentional selection [22]. The input and output specificities are the basis for the use of the many therapeutic drugs that mimic or antagonize the effects of the neurotransmitters from the ascending arousal system in different pathological conditions.

Additional support for a central role of histaminergic neurons in promoting arousal during a motivated behavior comes from experiments in mice with genetic alteration of the brain histaminergic system. Exploration of a new environment, also considered a motivated behavior [1], is consistently impaired in histidine decarboxylase K.O. mice [23] as well as in histamine H1 receptor K.O. mice [7]. It is compelling that histidine decarboxylase K.O. mice, comparable to TMN lesion animals [5], had normal 24-h locomotor activity, indicating a specific effect of the gene deficiency on exploration rather than a general effect on locomotor activity.

Our experiments with water deprivation, sexual drive or drug seeking behavior suggest a separation of the consummatory component from the appetitive behavior preceding rewards. The TMN showed increased Fos-ir only when water, receptive female or amphetamine seemed available to the rats. Two arousal nuclei, the LC and the DR showed increased Fos-ir in response to water deprivation and prior to enticing, confirming their sensitivity to negative water balance [24,25]. Their activation may be related to the known sensitivity of raphe neurons to brain metabolic challenges [21] and to the multisensory nature of locus coeruleus inputs [22].

Altogether, our findings support the notion that specific combinations of AAS nuclei activation underlie the arousal observed during defined appetitive behaviors and that the TMN always participates in these diverse motivated behaviors.

The role of brain histamine in drinking behavior remains controversial. Administration of histamine into the lateral hypothalamus increases water intake in water-satiated rats [26]. Histamine H1 or H2 receptor antagonists microinjected into the ventromedial hypothalamic nucleus reduce water intake [27], while their administration into the ventricles has no effect [28]. However, lesions restricted to either of TMN subdivisions E1-E4 induce a persistent polydipsia [29]. These contradictory results may be related to the different structures involved in those studies.

The relationship between histamine system and sexual behavior has been studied in females. The inhibition of histamine synthesis or the blockage of H1 histamine receptors decreases female copulatory responsiveness. [30]. Histamine depolarizes neurons in VMN (Ventromedial Nucleus), a key region in the hypothalamus that produces female sexual behavior and arousal, by modifying a leak potassium current on those neurons; this depolarization is enhanced in the presence of estradiol, a well-known estrogen that facilitates lordosis behavior [31,32]. While these results are in line with the present findings in male rats, more specific studies are needed to understand the contribution of the histamine system to sexual appetitive behavior in both sexes.

During the place preference test evaluating appetite for amphetamine, saline control animals showed no activation of the AAS. The animals that resisted the conditioning to amphetamine, and did not change their preference for the dark compartment in spite of receiving amphetamine, showed activation of the TMN and LC during the place preference test. However the animals conditioned to amphetamine showed a higher activation of TMN and, further, LHA/Orx neurons, DR and LDT were also engaged. The relationship of histaminergic system and drug addiction is still not clear, with contradictory evidence as has been discussed in recent reviews on this subject [33,34].

We have shown that increased arousal together with the early activation of the TMN are both present in four appetitive behaviors studied (drug-seeking, sexual motivation, appetite for food, and water-seeking), suggesting a role for histaminergic neurons in each of these motivated behaviors. This activation should be specific for motivation, because arousal *per se* is not always related to increased Fos-ir in the TMN, as seen in rats kept forcedly awake by tapping their cages [4]. In this passive awakening only the LC was active [4], as expected due to its known sensitivity to a wide range of intense or task-relevant sensory stimuli [22,35]. In the same vein, stimuli that increase vigilance, like amphetamine, LiCl injections, or restraint stress [36] failed to raise Fos-ir in the TMN.

In conclusion, lesion of the histaminergic neurons from the TMN provide novel evidence indicating that the histaminergic tuberomamillary nucleus is necessary for different motivated behavior including drinking, sexual drives and drug seeking. Histaminergic neurons are always an active, but not necessarily the only, component of the AAS that becomes excited during appetitive performance elicited by natural rewards, suggesting an important and specific role for histamine in the arousal that energizes and gives the proper intensity to these goal-directed behaviors.

## Supporting Information

S1 Fig Appetitive behaviors did not change Fos-ir expression in the VTA. (TIF)
