# Supplementary figures and images for: The Histaminergic Tuberomamillary Nucleus Is Involved in Appetite for Sex, Water and Amphetamine
Source: PLoS One. 2016 Feb 4;11(2):e0148484. doi: 10.1371/journal.pone.0148484 (PMC4743640; doi:10.1371/journal.pone.0148484)

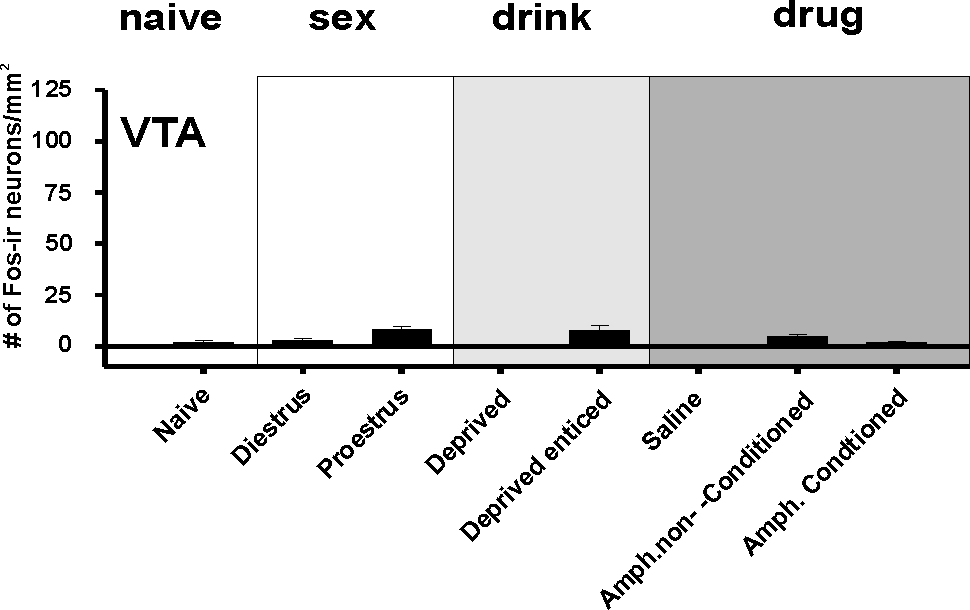

Supplement: S1 Fig — (TIF) [file pone.0148484.s001.tif]
